# Exploring Different Toxic Effects of UV-Aged and Bio-Aged Microplastics on Growth and Oxidative Stress of *Escherichia coli*

**DOI:** 10.3390/toxics13090706

**Published:** 2025-08-22

**Authors:** Juntong Gao, Qimeng Yang, Xiarui Fan, Xinwei Zhou, Peng Ren

**Affiliations:** 1 College of Environmental and Chemical Engineering, Jiangsu University of Science and Technology, Zhenjiang 212100, China; 2 School of Naval Architecture & Ocean Engineering, Jiangsu University of Science and Technology, Zhenjiang 212100, China; 3 College of Resources and Environmental Science, Nanjing Agricultural University, Nanjing 210095, China

**Keywords:** microplastics, *Escherichia coli*, physicochemical property, toxicity

## Abstract

Toxicological effects of microplastics (MPs) have been confirmed in a variety of microorganisms in aquatic environments, and they are closely correlated with the physicochemical properties of the MPs. In a natural environment, different aging treatments always induce different alterations in the physicochemical properties of MPs, thus influencing their environmental behaviors and biotoxicity. In this work, physicochemical properties and toxicity towards *Escherichia coli* (*E. coli*) were investigated in polystyrene (PS) MPs (3 and 10 μm) before and after aging by UV irradiation and biofilm formation, respectively. The results show that UV irradiation and biofilm formation led to different alterations in the surface morphologies and functional groups of PS. The UV-aged 3 μm PS had the strongest inhibitory effect on *E. coli* growth, and the bio-aged 10 μm PS had the strongest beneficial effect on *E. coli* growth. Also, the ATPase activity, production of intercellular ROS, and MDA content of the *E. coli* were affected differently. UV aging enhanced the toxicity of PS towards *E. coli*, while bio-aging had an opposite weakening effect. Overall, our research verified the remarkable differences in the physicochemical properties and biotoxicity of PS induced by UV aging and bio-aging.

## 1. Introduction

As the most widely used synthetic polymer materials, plastics have versatile applications in daily life due to their excellent properties, including their light weight, durability, and impact resistance [1]. In 2021, the global production of plastics dramatically increased to 390.7 million tons, which was approximately a 200-fold increase since 1950 [2]. Currently, most plastic waste is discarded, and the majority is likely to be released into natural environments [3,4]. Meanwhile, large plastic debris can be gradually degraded into microplastics (MPs), which are defined as plastic fragments or particles smaller than 5 mm in diameter [5,6]. As emergent pollutants, MPs occur ubiquitously in aquatic environments (marine environments, lakes, rivers, and other water bodies) and could pose stronger emerging risks to organisms than the original large pieces of plastic debris, which have been receiving increasing concern about their environmental risks [7,8]. Facing the threat of (micro)plastic contamination, various upstream and downstream responses have been carried out to prevent and tackle MP pollution, such as the circular economy, behavioral change, converting waste to energy, and plastic degradation [9,10].

Throughout the lifecycles of MPs, they inevitably undergo multiple aging processes driven by abiotic and biological factors, such as sunlight, oxygen, high temperatures, and active microorganisms, either simultaneously or sequentially [11,12,13]. These aging treatments alter the physicochemical characteristics of MPs (i.e., their morphologies, crystallinities, oxygen contents, molecular weights, and surface charges) and generate abundant secondary MPs, which further affect their fates, transport potentials, and environmental risks [14,15,16]. At present, MP aging is being studied under different exposure conditions.

Ultraviolet (UV) oxidation was verified as one of the fundamental causes of aged MP formations in aquatic systems [17]. UV aging induces C–H bond scission and forms peroxy radicals, thus generating oxygen-containing functional groups [18,19]. Furthermore, changes in surface morphology are demonstrated by observing surface roughness, cracks, and collapse [20,21]. In addition, crystallinity and hydrophobicity also evolve during UV aging. Many investigations have proved that UV aging induces MPs to be more active and favors further interactions with other environmental substances [22,23].

Bio-aging driven by biofilm formation is also a fundamental way for aged MPs to form in aquatic systems [14,24]. Microbial communities can readily colonize the surfaces of MPs entering an aquatic environment and form biofilms, which are referred to as the “plastisphere” [24,25]. The plastisphere could significantly affect the control of biogeochemical cycles, plastic biodegradation, and pathogenic activities [24,25]. Biofilms are mainly composed of various microorganisms (about 10% of the dry mass), as well as their self-produced extracellular polymeric substances (EPSs) (over 90% of the dry mass) [26,27]. Biofilm coverings on MP surfaces construct a cohesive, three-dimensional polymer layer as an interface in the plastisphere, which could potentially participate in interactions between MPs and allogenic microorganisms [28]. Biofilms also affect the surface physicochemical properties of MPs, including increased surface roughness, hydrophilicity, and oxidation contents [29,30,31,32]. It is worth noting that the effect of bio-aging on the physicochemical properties of MPs is very different from UV aging in terms of the strategies, pathways, and final performance of the MPs [33,34].

Recently, an increasing number of studies have also focused on interactions between MPs and microorganisms and have found that MPs exert certain toxic effects on microorganisms, which are manifested as inhibited microorganism growth and oxidative damage [35,36,37]. When microorganisms are experiencing oxidative stress, the accumulation of oxidative metabolites (ROS, MDA, etc.) in cells is closely associated with cell structure destruction and lipid damage, which are generally identified as the best indicators to assess the toxic effect mechanisms of micro-/nano-materials on microorganisms [15,38,39]. It has also been reported that long-term exposure to MPs can reduce ATPase activity due to changes in the cellular redox status and thus decreased energy supply [40,41].

Toxic effects of MPs on microorganisms are generally dependent on MP properties, including the particle size, surface characteristics, and polymer type, as well as the microorganism cell type [42,43,44]. For example, Ning [45] found that the toxicity of nano-PS was dependent on its size and functional modifications, and amino-modified PS (200 nm) showed the greatest toxicity, identified as a decrease in *E. coli* growth and a subsequent increase in intracellular ROS or probable mechanical damage. In addition, aging treatments for MPs also significantly influence their toxicity for microorganisms [35,46]. However, previous studies mostly focused on the influence of the properties of MPs themselves on their biotoxicity [42,43,44]. There is less research reporting the influence of the aging process on the biotoxicity of MPs [35,46]. Also, previous studies mainly focused on photo-aging [46,47], but the necessary knowledge about bio-aging (one of the most important aging processes) is lacking. Hence, more experimental studies are needed to further assess and compare the biological toxicity of MPs after different aging treatments driven by UV irradiation and biofilm formation.

Bacteria, as the foundation of aquatic ecosystems, are widely distributed in natural aquatic environments and are highly sensitive to environmental pollution [48,49]. Among them, *Escherichia coli* (*E. coli*), as a common model bacterium, is frequently detected on MP surfaces and is therefore utilized to test the toxicology of MPs [50]. In addition, polystyrene microparticles (PS MPs) were selected as model MPs because they are one of the most abundant types of plastic debris in the environment [51,52]. Therefore, the aim of this study was to investigate the differences between the effects of UV-aging and bio-aging treatments on the physicochemical properties of MPs and their toxicities towards *E. coli*, which could help to better assess the environmental behavior and potential risks of MPs.

## 2. Materials and Methods

### 2.1. Materials and Chemicals

Conventional PS MPs (defined as PS) with average particle sizes of 3 μm and 10 μm were purchased from Fengtai Polymer Materials Corporation (Dongguan, China). The conventional PS was processed by the manufacturer through ball grinding and primary sieving, without any functionalized treatment. KBr used in FTIR analysis was purchased from Aladdin Biochemical Technology Co., Ltd. (Shanghai, China). NaCl, Tryptone, and biotechnology-grade yeast extract were purchased from Sigma-Aldrich (Merck, Darmstadt, Germany) and used to prepare a Luria–Bertani (LB) medium for bacterial growth.

### 2.2. Preparation of Aged Microplastics

UV-aged PS MPs (defined as UV-PS) were prepared according to the modified methods in the literature [20,53,54]. Virgin PS was placed in quartz glass Petri dishes and continuously irradiated by two parallel UV lights at room temperature for 10 days. The 15 W UV lights had a primary wavelength of 254 nm and a radiant intensity of 15 W/m^2^. To ensure uniform UV exposure, the PS samples were mixed thoroughly twice a day.

To prepare bio-aged PS MPs (defined as Bio-PS), virgin PS was incubated in sampled freshwater under controlled laboratory conditions for 4 weeks to develop a biofilm on its surface. The freshwater was sampled from Lake Haiyun (32°06′37.36″ N, 119°21′48.01″ E). The water’s chemical properties are provided in Table S1. The biofilm development treatment was carried out according to the method in our previous study [31].

After the aging treatments, the UV-PS and Bio-PS were washed with Milli-Q water three times, dried at room temperature, and stored in glass bottles in the dark for future analysis.

### 2.3. Characterization of Virgin and Aged PS

The changes in the physicochemical properties of the PS were characterized before and after UV aging and bio-aging, including the surface morphology, functional groups, and hydrophobicity.

Firstly, to compare the alterations in the surface morphology, morphological characterization of the virgin PS, UV-PS, and Bio-PS was performed by scanning electron microscopy (SEM, Hitachi S-4800, Tokyo, Japan). Before the SEM observations, the samples needed to be dispersed on an electroconductive paste using tweezers and were then coated with platinum. The SEM was conducted at an electron-accelerating voltage of 5.00 kV.

Then, the changes in the functional groups of the virgin PS, UV-PS, and Bio-PS were investigated by Fourier transform infrared spectroscopy (FTIR). The spectrum scanning range was set from 4000 to 500 cm^−1^ with a resolution of 2 cm^−1^. The spectrum of each sample was detected by 32 scans with atmospheric correction. Based on the obtained FTIR spectra, the carbonyl index (CI) was calculated to further assess the aging degree of the MPs. According to a previous study [55], the CI was calculated as the ratio of the areas of the characteristic carbonyl peak (near 1720 cm^−1^) and the characteristic methylene peak band near 2960–2850 cm^−1^ (an internal constant band) for PS.

Furthermore, to observe the changes in hydrophobicity, the contact angles of the virgin PS, UV-PS, and Bio-PS were measured using an optical contact angle goniometer (SPCA, Beijing Hake Test Instrument Factory, Beijing, China). In addition, the biomass on the Bio-PS was quantitatively determined by the Fenton oxidation method [31].

### 2.4. Bacteria Cultivation and Exposure

Gram-negative *Escherichia coli (E. coli DH5α*) was applied as a model bacteria to evaluate the toxic effects of virgin PS, UV-PS, and Bio-PS on bacteria. The *E. coli* strain was revived in 5 mL of LB medium at 37 °C and 150 rpm for 24 h, then transferred into 1 L of fresh LB medium and cultured for another 12 h to reach the logarithmic phase. The bacteria were separated from the LB medium by centrifugation (6000× *g* at 4 °C) for 10 min, then were washed with Milli-Q water three times. The obtained cell pellets were resuspended with a 10-fold-diluted LB medium in Milli-Q water until the optical density at 600 nm reached approximately 0.02 for the MP exposure experiment.

To initiate the batch exposure experiments, different aliquots of virgin PS, UV-PS, and Bio-PS with two particle sizes were added to the bacterial suspension in priorly sterilized 100 mL glass vials equipped with polytetrafluoroethylene caps. The initial concentrations of the MPs were 20, 100, and 200 mg/L. A batch exposure experiment without any PS-MPs was used as a control group. The samples were cultured at 37 °C and 150 rpm/min for 24 h, and aliquots (<1.0 mL) were sampled at desired time intervals (0–36 h). The growth curves were measured by the optical density of each culture medium at 600 nm (OD600) at intervals of 0 to 36 h using a UV–vis spectrometer, according to previous studies [35,44]. The experiments were carried out in triplicate unless otherwise mentioned. The normalized growth ratio (*NGR*) was calculated by the following equation:

(1) $$NGR\%=\frac{OD_{i}}{OD_{0}}\times 100\%$$

where *OD*_0_ and *OD_i_* represent the *OD* readings of the control and treated groups, respectively.

### 2.5. Determination of ATPase Activity, Production of ROS, and MDA Content

The differences in the physiological mechanisms of *E. coli* under the stresses of PS before and after UV aging and bio-aging were assessed. After exposure to 200 mg/L PS, UV-PS, or Bio-PS for 3 h or 24 h, the ATPase activity, production of ROS, and MDA activity of the *E. coli* were measured using diagnostic reagent kits (Nanjing Jiancheng Bioengineering Institute, Nanjing, China), following the manufacturer’s detailed instructions.

To determine the ATPase activity, a fresh *E. coli* cell suspension was centrifuged at 5000 rpm, washed three times, and resuspended in a phosphate-buffered saline (PBS) solution. The homogenates were prepared by pulverizing them with an ultrasonic pulverizer. The ATPase activity was detected by an ultraviolet spectrophotometer at 636 nm, according to the instructions of the Ultramicro ATPase Activity Detection Kit.

The intracellular generation of ROS in the *E. coli* cells was detected using the fluorescent probe 20,70-dichlorofluorescin diacetate (DCFH-DA). An *E. coli* cell suspension was centrifuged three times. Then, 10 mM DCFH-DA was added to the cell pellet to prepare a cell suspension. The cells were incubated at 37 °C for 30 min in the dark using a shaking incubator. They were then washed three times with a PBS solution and were finally suspended to obtain a supernatant. The fluorescence of the supernatant was measured using a fluorescence spectrophotometer.

The intracellular MDA content was also detected. Similar to the ATPase assay, a cell homogenate was prepared, mixed with a thiobarbituric acid solution, and heated in a water bath for 40 min. After heating, the supernatant was cooled and centrifuged at 3500 rpm for 10 min. The absorbance value of the supernatant was measured at 532 nm by a UV–vis spectrometer.

### 2.6. Statistical Analysis

The experimental data were expressed as means ± SDs, and a one-way analysis of variance (ANOVA) was conducted with a Least Significant Difference (LSD) post hoc test. The levels of significance were set at *p* < 0.05 (*), *p* < 0.01 (**), and *p* < 0.001 (***).

## 3. Results and Discussion

### 3.1. Changes in Physicochemical Properties of Virgin and Aged PS

The different aging treatments could induce various changes in the physicochemical properties of PS and further influence the toxic effect of PS on bacteria [36,37]. Comparing the multiple characterizations of virgin PS, UV-PS, and Bio-PS showed that the different aging treatments driven by UV irradiation and biofilm formation significantly altered the physicochemical characteristics of the PS, including the surface morphology, functional groups, and hydrophobicity.

#### 3.1.1. Surface Morphology

**Figure 1:**
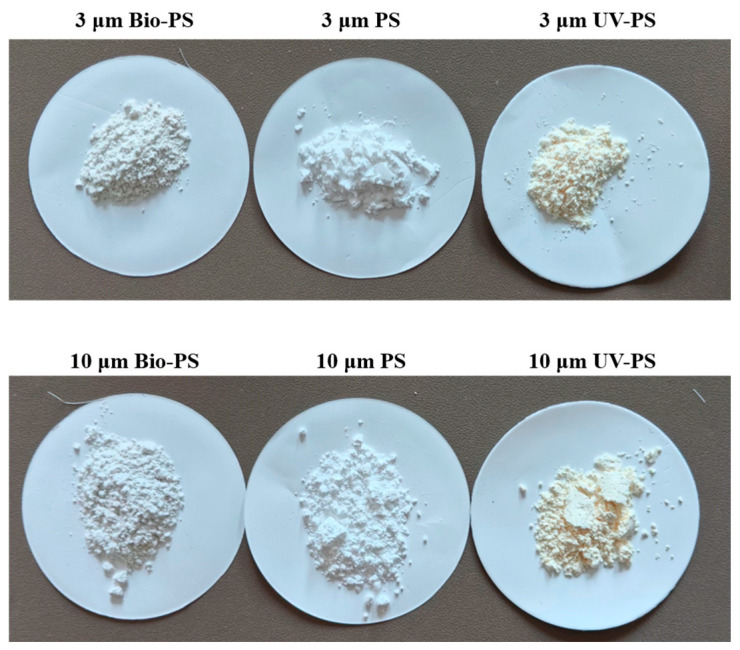
Images of 3 μm and 10 μm PS, UV-PS, and Bio-PS.

As shown in Figure 1, after 10 days of UV irradiation, the external color of the PS changed from white to light yellow, suggesting the generation of oxygen-containing moieties in the UV-PS. Meanwhile, after 30 days of freshwater incubation, the external color of the Bio-PS changed to light greyish green, which was consistent with observations by the naked eye in studies by Tu et al. [12] and Gao et al. [31].

**Figure 2:**
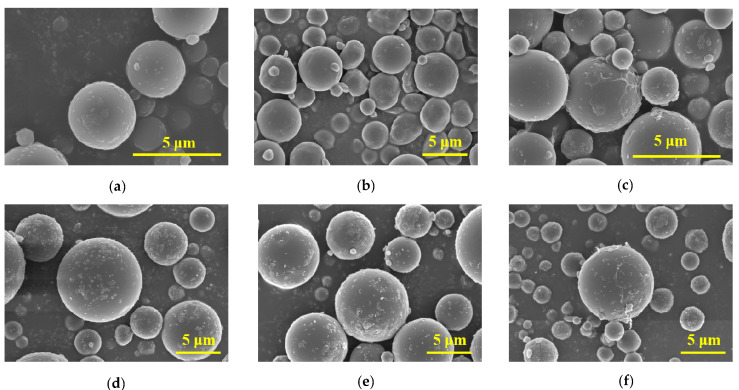
SEM images of 3 and 10 μm PS, UV-PS, and Bio-PS: (**a**) 3 μm PS; (**b**) 3 μm UV-PS; (**c**) 3 μm Bio-PS; (**d**) 10 μm PS; (**e**) 10 μm UV-PS; (**f**) 10 μm Bio-PS.

The microscopic morphologies of the PS, UV-PS, and Bio-PS were characterized by SEM analysis, as shown in Figure 2a–f. Based on the magnified SEM images, the virgin PS was an approximately spherical particle. The 3 μm PS exhibited a relatively smooth surface with a few small protrusions that were probably caused by ball grinding damage. The surface morphology of the 10 μm PS was similar to that of the 3 μm PS, but it exhibited more protrusions. Moreover, the particle sizes of the virgin PS were not uniform, as shown in Figure S1. By comparison, after the UV-aging treatment, the surface morphology of the 3 μm PS changed to be more irregular, and large numbers of wrinkles, fractured surface textures, and grooves were observed, verifying that significant alterations in the surface morphology were induced by UV aging. Moreover, some secondary particles with smaller sizes were also observed. Compared with the 3 μm UV-PS, the surface morphology of the 10 μm UV-PS was not obviously changed, although more protrusions and wrinkles were observed compared with the virgin PS. The surface morphology of the Bio-PS was smoother with microsphere aggregation, which was significantly different from the virgin PS and UV-PS. These distinctive features were assigned to the formed biofilms, which were composed of adhering bacteria and dense layers of EPSs [56].

#### 3.1.2. Functional Groups

**Figure 3:**
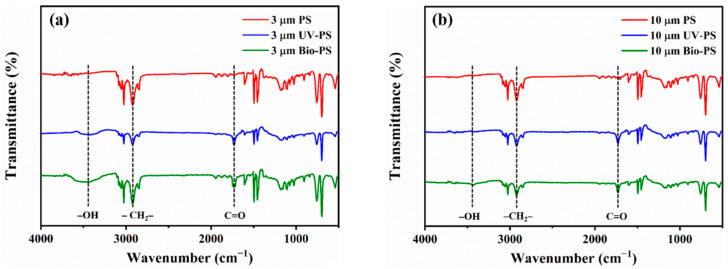
FTIR images of 3 and 10 μm PS, UV-PS, and Bio-PS: (**a**) 3 μm PS, UV-PS, and Bio-PS; (**b**) 10 μm PS, UV-PS, and Bio-PS.

To explore the changes in the PS structure caused by UV aging and bio-aging, FTIR spectroscopy was applied to determine the functional group changes before and after aging. The FTIR spectra of the virgin PS, UV-PS, and Bio-PS are shown in Figure 3. Compared with the virgin PS, stronger peaks and additional peaks characteristic of -OH and C=O were observed at 3438 and 1726 cm^−1^ in the UV-PS and Bio-PS, indicating the generation of oxygen-containing functional groups. The CI values of the 3 and 10 μm UV-PS were 0.49 and 0.87, and the CI values of the 3 and 10 μm Bio-PS were 0.35 and 0.58, respectively. These results show that the CI values of the UV-PS were significantly higher than those of the virgin PS and Bio-PS, suggesting that the UV-PS exhibited the highest oxidation degree. The oxidation mechanisms of the UV-PS and Bio-PS were different. The oxidation of the UV-PS was attributed to free radical generation and chain scissions caused by UV irradiation, but the oxidation of the Bio-PS was mainly derived from EPSs secreted by microorganisms and natural organic matter adhering to the MP surfaces [12,24,31].

#### 3.1.3. Hydrophobicity

**Figure 4:**
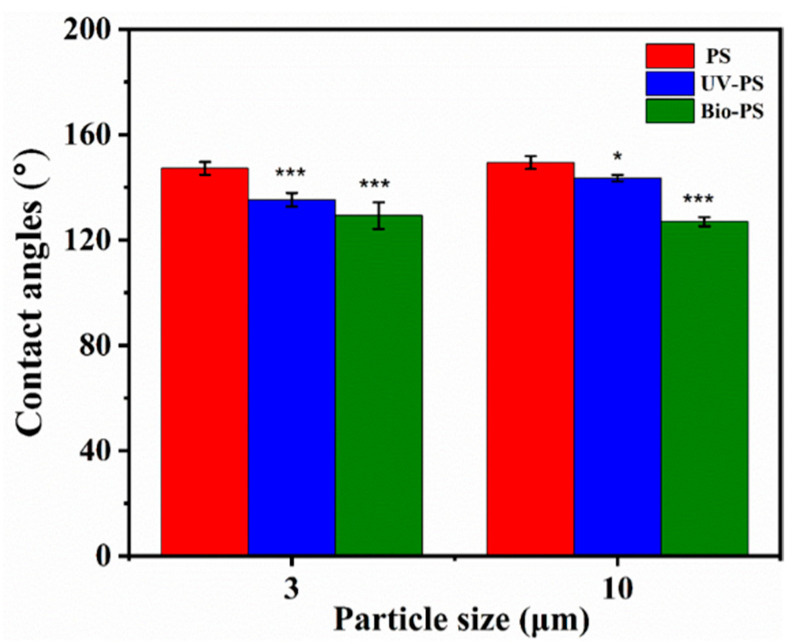
The water contact angles on the surfaces of the 3 and 10 μm PS, UV-PS, and Bio-PS. Asterisks (*) indicate statistically significant differences compared to the control group. The levels of significance were set at *p* < 0.05 (*), and *p* < 0.001 (***).

The water contact angle is widely used as an indicator of a material’s hydrophobicity (s), which is closely related to changes in functional groups. A greater contact angle indicates stronger hydrophobicity for the tested material. Figure 4 shows that all aged PS samples showed smaller water contact angles compared to the 3 and 10 μm virgin PS (147.2° and 149.4°). The water contact angles of the 3 and 10 μm UV-PS decreased by 8.1% and 4.0%, respectively, compared with the virgin PS. Similarly, the 3 and 10 μm Bio-PS showed greater decreases of 12.2% and 15.0%, respectively. These results demonstrate that the Bio-PS exhibited significantly lower hydrophobicity than the UV-PS because the biofilm covering the MP surfaces formed an interface layer containing abundant hydrophilic functional groups [12,26].

#### 3.1.4. Fouling Biomass of Bio-PS

The fouling biomass on the surfaces was quantitatively determined by modified Fenton oxidation. After 4 weeks of incubation, the amounts of fouling biomass on the 3 and 10 μm Bio-PS reached 34.3% and 41.2% of the total masses of Bio-PS, respectively. The amount of biomass on the 10 μm Bio-PS was 1.2-fold higher than that on the 3 μm Bio-PS, indicating that the PS with a larger size was more conducive to the colonization of microorganisms and the formation of biofilms on MP surfaces.

### 3.2. Effects of Virgin and Aged PS on Cell Growth of E. coli

A growth curve was applied to investigate the effects of 3 and 10 μm virgin PS, UV-PS, and Bio-PS on *E. coli* growth, as shown in Figure S2. As a whole, exposure to virgin PS, UV-PS, and Bio-PS influenced *E. coli* growth to various extents compared to the control group, and their influences were closely related to the properties of the PS, including the aging treatment, exposure concentration, and particle size. To further clarify the effect of PS on *E. coli* growth, the normalized growth ratio was calculated and compared.

#### 3.2.1. Effects of Virgin PS on Cell Growth of *E. coli*

**Figure 5:**
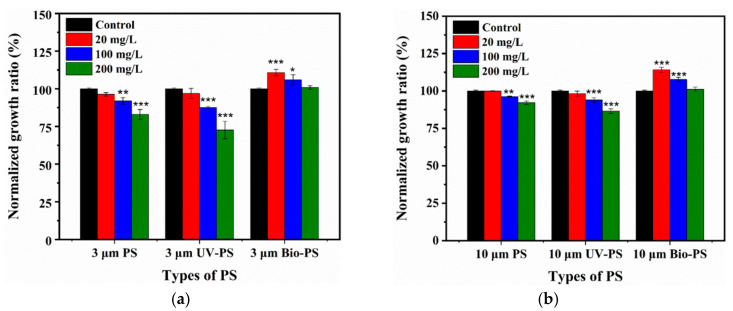
The normalized growth ratios of *E. coli* exposed to 3 and 10 μm PS, UV-PS, and Bio-PS: (**a**) 3 μm PS, UV-PS, and Bio-PS; (**b**) 10 μm PS, UV-PS, and Bio-PS. Asterisks (*) indicate statistically significant differences compared to the control group. The levels of significance were set at *p* < 0.05 (*), *p* < 0.01 (**), and *p* < 0.001 (***).

As shown in Figure 5a, the 3 μm virgin PS had a negligible inhibitory effect on *E. coli* growth at 20 mg/L (*p* > 0.05). At 100 and 200 mg/L, the 3 μm virgin PS significantly inhibited *E. coli* growth by 7.1% and 17.0% (*p <* 0.05). As shown in Figure 5b, the 10 μm virgin PS also inhibited *E. coli* growth in a dose-dependent manner from 0.1% at 20 mg/L to 7.8% at 200 mg/L.

The virgin PS inhibited *E. coli* growth in both size- and dose-dependent manners, where a smaller size and a higher exposure concentration demonstrated stronger toxicity. These results were similar to previous research findings [57,58]. For example, PS debris with smaller particles induced greater reductions in the viability of *Halomonas alkaliphile*, with stronger toxicity, highlighting the size-dependent effect [57]. Similar results were reported by Liu [58], who showed that the toxic effects of PS particles towards *Scenedesmus obliquus* were negatively correlated with the particle size (100–2000 nm), with 100 nm PS showing the strongest toxicity.

#### 3.2.2. Effects of UV-PS on Cell Growth of *E. coli*

Furthermore, UV-PS and Bio-PS were applied to assess the effects of different aging treatments on the biotoxicity of PS. At 20 mg/L, both the 3 and 10 μm UV-PS had slight inhibitory effects on the *E. coli* growth ratio, 3.1% and 1.7% (*p* > 0.05), similar to the virgin PS. However, considerable declines in the *E. coli* growth ratio (*p <* 0.05) were observed for the 3 and 10 μm UV-PS at 100 and 200 mg/L. Among them, the 3 and 10 μm UV-PS resulted in the strongest inhibition of *E. coli* growth (27.4% and 13.4%) at 200 mg/L. These inhibition values were significantly higher than those of the virgin PS (17.0% and 7.8%). Moreover, the inhibitory effect of the UV-PS was positively correlated with the exposure concentration, similar to the virgin PS.

Overall, the UV-PS demonstrated a significantly stronger inhibitory effect on *E. coli* growth compared to the virgin PS with an identical particle size at a higher exposure concentration. The present results suggest that UV aging played an important role in enhancing the toxic effect of PS on *E. coli* growth. They are also supported by a previous study reporting that the inhibitory effects of styrene–butadiene–rubber MPs with different UV-aging degrees on bacterial growth were strengthened to different extents when compared with virgin MPs. This was because the changes in the surface characteristics and production of smaller MP particles enhanced their interactions with bacteria [35,46].

#### 3.2.3. Effects of Bio-PS on Cell Growth of *E. coli*

Unlike the virgin PS and UV-PS, the Bio-PS did not cause any inhibition of *E. coli* growth. In contrast, exposure to the Bio-PS enhanced *E. coli* growth to different extents. When exposed to 3 μm Bio-PS at 20 mg/L, *E. coli* growth was significantly enhanced by 10.8% relative to the control group. However, when the exposure concentration increased to 100 and 200 mg/L, the enhancement effects of the 3 μm Bio-PS on *E. coli* growth were weakened, with enhancement ratios of 5.9% and 0.9%, respectively. When exposed to the 10 μm Bio-PS at 20, 100, and 200 mg/L, *E. coli* growth was further enhanced by 14.2%, 7.7%, and 1.2%, respectively, which was superior to exposure to the 3 μm Bio-PS. These results indicate that the enhancement effects of the Bio-PS were negatively correlated with the exposure concentration but positively correlated with the particle size, which was completely opposite to the inhibitory effect of the virgin PS and UV-PS.

Therefore, biofilm formation on MP surfaces after bio-aging could effectively weaken the toxic effects of MPs on *E. coli* and even significantly promote *E. coli* growth when exposed to low concentrations and large particle sizes. This is because biofilm coatings on MP surfaces could impede contact and interactions between MP surfaces and bacteria, thereby significantly weakening the toxicity of MPs to *E. coli*. Moreover, as important components of biofilms, EPSs play a crucial role in biofilm cohesion by enabling stable adhesion to MP surfaces and providing more varieties of active reaction sites [14,59]. EPSs include various soluble substances such as hydrophilic polysaccharides, humic acid, and amino acids [59,60]. Hence, EPSs could serve as a carbon source to provide rich nutrients for *E. coli* growth. Previous studies reported that biofilm coatings on MP surfaces could potentially block UV aging [28], which verified the role of the biofilm layer as an interface barrier to mediate interactions between MPs and surrounding environmental factors.

### 3.3. Effects of Virgin and Aged PS on ATPase Activity

**Figure 6:**
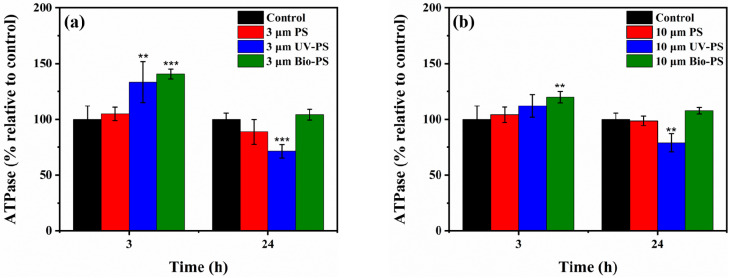
The relative ATPase activity of *E. coli* exposed to 3 and 10 μm PS, UV-PS, and Bio-PS: (**a**) 3 μm PS, UV-PS, and Bio-PS; (**b**) 10 μm PS, UV-PS, and Bio-PS. Asterisks (*) indicate statistically significant differences compared to the control group. The levels of significance were set at *p* < 0.01 (**), and *p* < 0.001 (***).

Figure 6 shows the effects of virgin PS, UV-PS, and Bio-PS on *E. coli* ATPase activity relative to the control group after 3 h and 24 h of exposure. After exposure to the virgin PS, regardless of the exposure time and particle size, the *E. coli* ATPase activity was similar to that of the control group, indicating that the virgin PS had a negligible influence on the *E. coli* ATPase activity (*p* > 0.05). Different from the virgin PS, both the UV-PS and Bio-PS impacted the *E. coli* ATPase activity to different extents. At 3 h, the UV-PS and Bio-PS significantly stimulated *E. coli* ATPase activity (*p <* 0.05), except for the 10 μm UV-PS. Among them, the 3 μm Bio-PS had the most potent enhancement effect on the *E. coli* ATPase activity, with an enhancement ratio of 40.6% compared with the control group. The above results indicate that a short period of exposure to aged PS could stimulate ATPase activity. This may be due to the different surface characterizations of aged PS, which caused *E. coli* to present a strong self-protective ability and strengthened its cellular defense and adaptive response to stress [61].

However, after 24 h of exposure, the UV-PS and Bio-PS showed different effects on *E. coli* ATPase activity. Regardless of whether the particle size of the MPs was 3 μm or 10 μm, long-term collisions between UV-PS and *E. coli* led to significant decreases in ATPase activity (28.5% and 21.0%) compared with the control group, especially for the small-sized UV-PS, suggesting that UV-PS could weaken ATPase activity and that the effect of small-sized MPs is more significant. The decreased ATPase activity negatively affected cell reproduction, as evidenced by the high inhibition rates of the virgin PS and UV-PS for *E. coli* growth. It has been reported that changes in the cellular redox status originating from exposure to MPs might cause a reduction in ATPase activity and thus reduce the energy supply [40,41]. In contrast, after long-term exposure to the Bio-PS, the *E. coli* ATPase activity did not show significant changes compared with the control group. It was found that *E. coli* could coexist with the Bio-PS after a short-term stress response (3 h), followed by *E. coli* ATPase activity returning to the normal level at 24 h.

### 3.4. Effects of Virgin and Aged PS on Oxidative Stress

**Figure 7:**
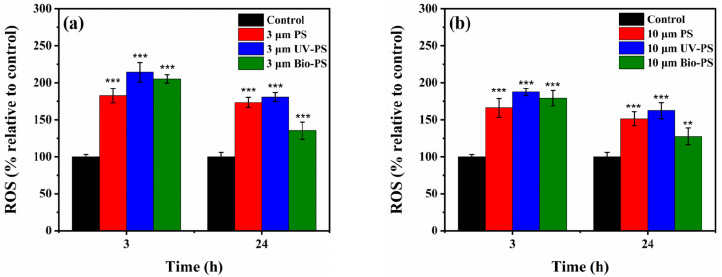
The relative ROS contents of *E. coli* exposed to 3 and 10 μm PS, UV-PS, and Bio-PS: (**a**) 3 μm PS, UV-PS, and Bio-PS; (**b**) 10 μm PS, UV-PS, and Bio-PS. Asterisks (*) indicate statistically significant differences compared to the control group. The levels of significance were set at *p* < 0.01 (**), and *p* < 0.001 (***).

The intracellular ROS generated by *E. coli* after exposure to virgin PS, UV-PS, and Bio-PS were detected, aiming to further clarify the toxic mechanisms by which different types of PS affect *E. coli*. As shown in Figure 7, after 3 h of exposure to 3 and 10 μm virgin PS, the production of intracellular ROS significantly increased by 82.5% and 65.9%, respectively, compared to the control group (*p <* 0.05). The 3 μm virgin PS had a stronger enhancement effect on the intracellular ROS level because small-sized MPs with greater surface areas have more opportunities to interact with bacterial cells [43]. Compared with the virgin PS, short-term exposure to the UV-PS and Bio-PS stimulated the *E. coli* to produce more intracellular ROS. Regardless of the particle size, when exposed to the UV-PS, the intracellular ROS production (214.0% and 187.4% compared to the control group) was higher than when exposed to the Bio-PS. These results indicate that after UV aging and bio-aging the PS elicited stronger stress responses in bacterial cells, with greater toxicity after short-term exposure.

After long-term exposure (24 h) to the 3 and 10 μm virgin PS, the production of intracellular ROS (205.0% and 179.1%) was still significantly higher than that in the control group (*p <* 0.05). When exposed to the UV-PS, the intracellular ROS level was not significantly different from that exposed to the virgin PS (*p* > 0.05). However, when exposed to the Bio-PS, the intracellular ROS levels (135.3% and 127.3%) in the *E. coli* cells were significantly lower than those in the cells exposed to the virgin PS and UV-PS (*p* < 0.05). It can be seen that the production of intracellular ROS remained at high levels after long-term exposure to the virgin PS and UV-PS, but the production of intracellular ROS induced by the Bio-PS was lower. This result suggests that bio-aging effectively weakened the stress responses in the cells with weaker toxicity, owing to the biofilm coatings on the Bio-PS surfaces impeding interactions with *E. coli* cells. The changes in the intracellular ROS levels were consistent with the growth curves of the *E. coli* upon exposure to the virgin PS and PS aged by UV irradiation and biofilm formation.

**Figure 8:**
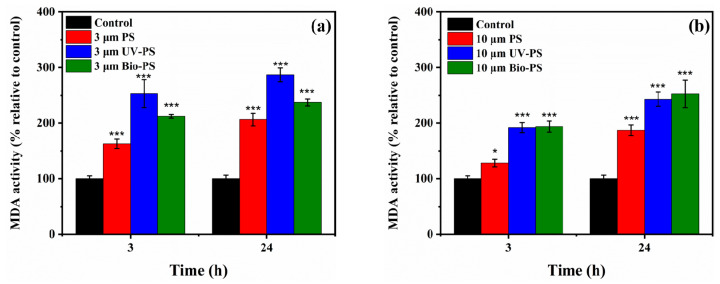
The relative MDA contents of *E. coli* exposed to 3 and 10 μm PS, UV-PS, and Bio-PS: (**a**) 3 μm PS, UV-PS, and Bio-PS; (**b**) 10 μm PS, UV-PS, and Bio-PS. Asterisks (*) indicate statistically significant differences compared to the control group. The levels of significance were set at *p* < 0.05 (*), and *p* < 0.001 (***).

The MDA levels relative to the control group are presented in Figure 8. After short-term exposure to the virgin PS, UV-PS, and Bio-PS, the relative MDA levels in the *E. coli* significantly increased compared to the control group (*p <* 0.05). Upon exposure to the 3 μm UV-PS, the relative MDA level (253.1%) in the *E. coli* was the highest. When the exposure time was 24 h, the change trends of the relative MDA level were similar to those after an exposure time of 3 h, and they remained at relatively high levels compared to the control group. Exposure to the virgin PS, UV-PS, and Bio-PS resulted in serious lipid damage occurring in *E. coli* cells, and the smaller particles exhibited a stronger enhancement effect on the MDA levels, indicating stronger toxicity towards *E. coli*. Moreover, the changes in the relative MDA levels were consistent with the production of intercellular ROS, verifying the correlative changes in oxidative stress.

On the whole, exposure to the virgin PS, UV-PS, and Bio-PS mostly stimulated the *E. coli* to inhibit ATPase activity, produce ROS, and increase the MDA content to significantly different extents (*p* < 0.05, Tables S2–S4), indicating that the various MP types had different toxicities towards *E. coli*, which may have been due to the different treatments used to age the PS.

## 4. Conclusions

In this study, multiple characterization methods were used to analyze changes in the physicochemical properties of PS before and after UV-aging and bio-aging treatments. It was found that after the UV-aging and bio-aging treatments the PS exhibited different physicochemical properties in terms of its surface topography, oxygen-containing functional groups, and hydrophobicity. The different alterations of the physicochemical characterizations driven by UV irradiation and biofilm formation influenced the toxicity of the PS to different extents and even reversed it. The virgin PS showed a certain toxicity to *E. coli* at the higher exposure concentration. The UV-aging treatment enhanced the toxic effect of PS on *E. coli* compared with the virgin PS. This manifested as a higher growth inhibition ratio and stronger oxidative stress, which was closely related to the liberation of smaller MP particles and adverse changes in physicochemical properties. In contrast to the UV-aging treatment, the bio-aging treatment induced biofilms as interface layers coating MP surfaces. This hindered interactions with *E. coli* cells and provided a nutrient source for *E. coli* growth. Hence, the bio-aging treatment enhanced *E. coli* growth, as it attenuated oxidative stress. Our results provide a basis for assessing the different changes in the physicochemical properties of PS after UV aging and bio-aging, as well as their toxic impacts on *E. coli* growth, ATPase activity, and oxidative stress, which is conducive to evaluating the potential risks of PS undergoing the complex aging process in freshwater ecosystems.

## Data Availability

The original contributions presented in this study are included in the article/Supplementary Material. Further inquiries can be directed to the corresponding authors.

## Supplementary Materials

The following supporting information can be downloaded at https://www.mdpi.com/article/10.3390/toxics13090706/s1, Figure S1: The particle size distributions of 3 μm and 10 μm PS and UV-PS calculated using SEM spectra and Image J software; Figure S2: Growth curve of *E. coli* exposed to 3 μm and 10 μm PS, UV-PS, and Bio-PS: (a) 3 μm PS, (b) 3 μm UV-PS, (c) 3 μm Bio-PS, (d) 10 μm PS, (e) 10 μm UV-PS, and (f) 10 μm Bio-PS; Table S1: Key water chemistry parameters of sampled freshwater; Table S2: The significance levels of *E. coli* ATPase activity after 24 h of exposure to 3 μm and 10 μm virgin PS, UV-PS, and Bio-PS; Table S3: The significance levels of intracellular ROS production after 24 h of exposure to 3 μm and 10 μm virgin PS, UV-PS, and Bio-PS; Table S4: The significance levels of the MDA content after 24 h of exposure to 3 μm and 10 μm virgin PS, UV-PS, and Bio-PS. References [62,63,64] are cited in the supplementary materials.
