# Supplementary material for: Exploring Different Toxic Effects of UV-Aged and Bio-Aged Microplastics on Growth and Oxidative Stress of Escherichia coli
Source: Toxics. 2025 Aug 22;13(9):706. doi: 10.3390/toxics13090706 (PMC12473941; doi:10.3390/toxics13090706)
Supplement: Supplementary file 1 [file toxics-13-00706-s001.zip › toxics-3822503-supplementary.pdf]

Exploring Different Toxic Effects of UV-Aged and Bio-Aged Microplastics on Growth and Oxidative Stress of *Escherichia coli*

Juntong Gao [1], Qimeng Yang [1], Xiarui Fan [2], Xinwei Zhou [1,3,*] and Peng Ren [2,*]

[1] College of Environmental and Chemical Engineering, Jiangsu University of Science and Technology, Zhenjiang, 212100, China
[2] School of Naval Architecture & Ocean Engineering, Jiangsu University of Science and Technology, Zhenjiang, 212100, China
[3] College of Resources and Environmental Science, Nanjing Agricultural University, Nanjing, 210095, China
* Correspondence: zhouxinwei@just.edu.cn (X.Z.); renpeng@just.edu.cn (P.R.)

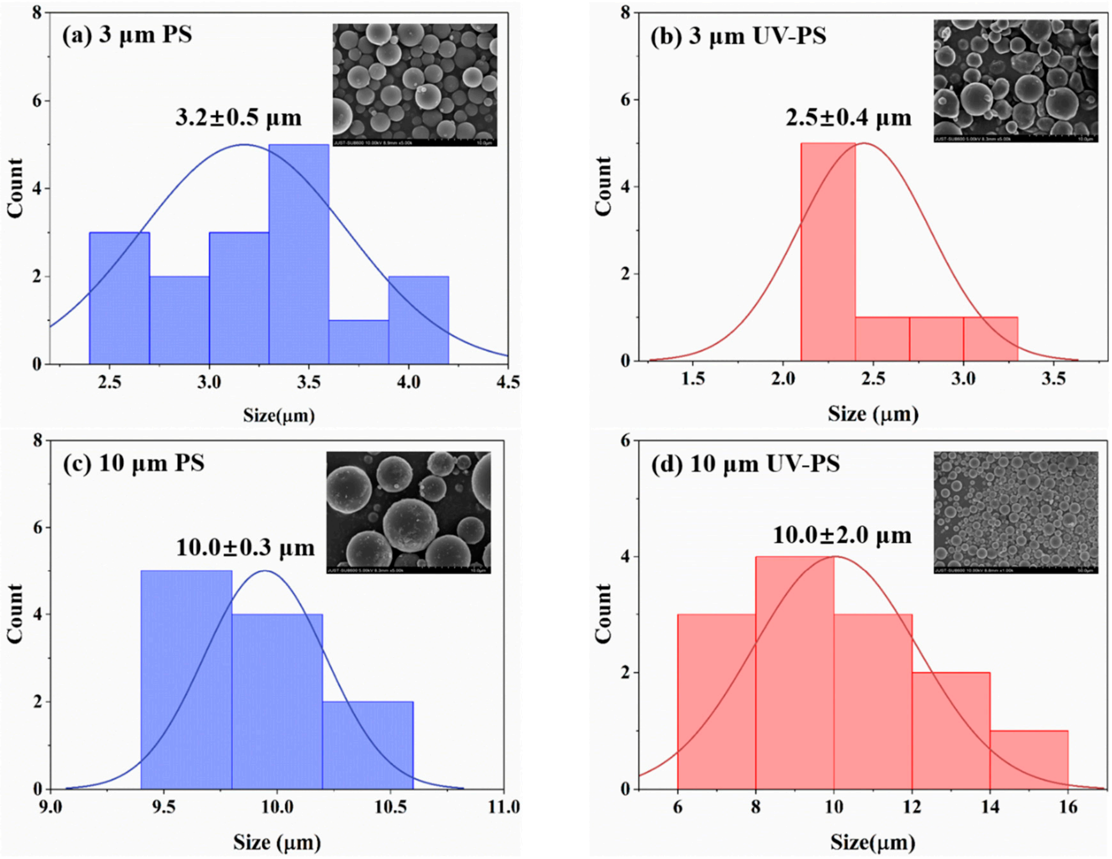

Figure S1. The particle size distributions of 3 μm and 10 μm PS, UV-PS calculated by using SEM spectra and Image J software.

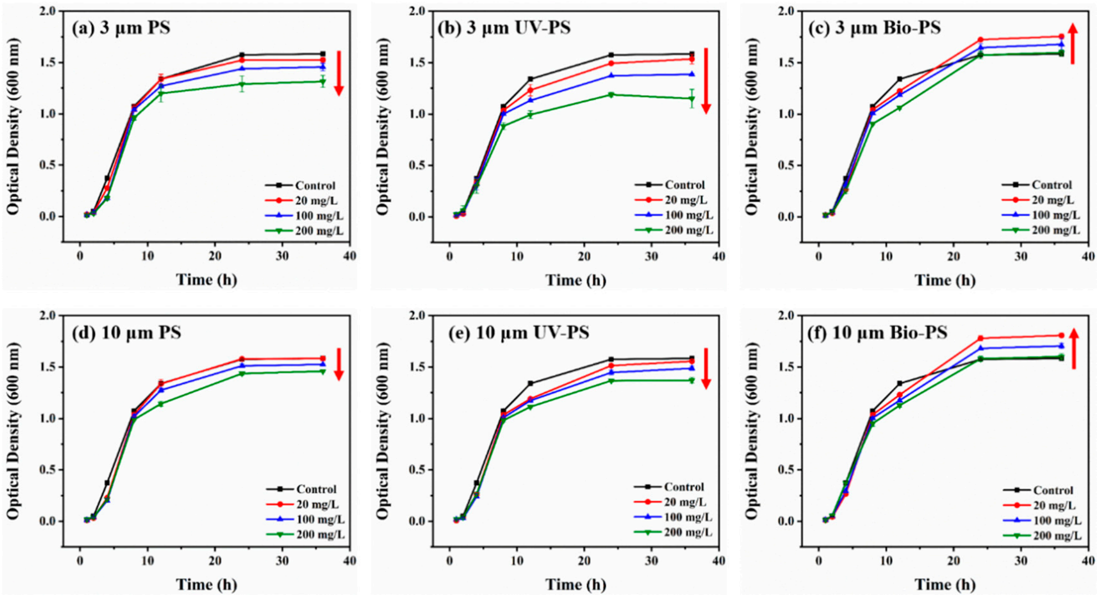

Figure S2. Growth curve of *E. coli* exposed to 3 μm and 10 μm PS, UV-PS and Bio-PS. (a), (b) and (c) 3 μm PS, UV-PS and Bio-PS; (d), (e) and (f) 10 μm PS, UV-PS and Bio-PS.

Table S1. Key water chemistry parameters of the sampled freshwater.

Parameters	pH (/)	DOC (mg/L)	TN (mg/L)
Freshwater	7.63±0.06	3.73±0.50	1.99±0.18

Key water chemistry parameters of the sampled freshwater were analyzed, including pH, dissolved organic carbon (TOC), and total nitrogen (TN), following the methods from previous studies [62–64]. Data were presented as Means ± SDs, n=3.

Table S2. The significance levels of *E. coli* ATPase activity at 24 h exposure to 3 μm and 10 μm virgin PS, UV-PS and Bio-PS. The significance level is set as $p > 0.05$ (−), $p < 0.05$ (*), $p < 0.01$ (**), and $p < 0.001$ (***). (The red and blue signs represent different types of PS with particle sizes of 3 μm and 10 μm, respectively).

	Virgin PS	UV-PS	Bio-PS
Control	–<br>–	$p < 0.001$ (***)<br>$p < 0.01$ (**)	–<br>–
Virgin PS		$p < 0.05$ (*)<br>$p < 0.01$ (**)	$p < 0.05$ (*)<br>–
UV-PS			$p < 0.001$ (***)<br>$p < 0.001$ (***)

Table S3. The significance levels of the intracellular ROS production at 24 h exposure to 3μm and 10μm virgin PS, UV-PS and Bio-PS. The significance level is set as $p > 0.05$ (−), $p < 0.05$ (*), $p < 0.01$ (**), and $p < 0.001$ (***). (The red and blue signs represent different types of PS with particle sizes of 3 μm and 10 μm, respectively).

	Virgin PS	UV-PS	Bio-PS
Control	$p < 0.001$ (***)<br>$p < 0.001$ (***)	$p < 0.001$ (***)<br>$p < 0.001$ (***)	$p < 0.001$ (***)<br>$p < 0.01$ (**)
Virgin PS		–<br>–	$p < 0.001$ (***)<br>$p < 0.01$ (**)
UV-PS			$p < 0.001$ (***)<br>$p < 0.001$ (***)

Table S4. The significance levels of MDA content at 24 h exposure to 3 μm and 10 μm virgin PS, UV-PS and Bio-PS. The significance level is set as $p > 0.05$ (−), $p < 0.05$ (*), $p < 0.01$ (**), and $p < 0.001$ (***). (The red and blue signs represent different types of PS with particle sizes of 3μm and 10μm, respectively).

	**Virgin PS**	**UV-PS**	**Bio-PS**
Control	$p < 0.001$ (***)<br>$p < 0.001$ (***)	$p < 0.001$ (***)<br>$p < 0.001$ (***)	$p < 0.001$ (***)<br>$p < 0.001$ (***)
Virgin PS		$p < 0.001$ (***)<br>$p < 0.001$ (***)	$p < 0.01$ (**)<br>$p < 0.001$ (***)
UV-PS			$p < 0.001$ (***)<br>–

References

62.Bekiari, V.; Avramidis, P. Data Quality in Water Analysis: Validation of Combustion-infrared and Combustion-chemiluminescence Methods for the Simultaneous Determination of Total Organic Carbon (TOC) and Total Nitrogen (TN). *Int. J. Environ. Anal. Chem.* **2013**, *94*, 65–76.
63.Roig, B.; Gonzalez, C.; Thomas, O. Simple UV/UV-visible Method for Nitrogen and Phosphorus Measurement in Wastewater. *Talanta* **1999**, *504*, 751–758.
64.Rozman, U.; Filker, S.; Kalčíková, G. Monitoring of Biofilm Development and Physico-chemical Changes of Floating Microplastics at the Air-water Interface. *Environ. Pollut.* **2023**, *322*, 121157.
